# Polyphyllin II inhibits liver cancer cell proliferation, migration and invasion through downregulated cofilin activity and the AKT/NF-κB pathway

**DOI:** 10.1242/bio.046854

**Published:** 2020-02-07

**Authors:** Dejiang Pang, Chengcheng Yang, Chao Li, Yuanfeng Zou, Bin Feng, Lixia Li, Wentao Liu, Qihui Luo, Zhengli Chen, Chao Huang

**Affiliations:** 1 Laboratory of Experimental Animal Disease Model, College of Veterinary Medicine, Sichuan Agricultural University, Chengdu 611130, China; 2 Neuroscience & Metabolism Research, State Key Laboratory of Biotherapy, West China Hospital, Sichuan University and Collaborative Innovation Center, Chengdu 610041, China; 3 Key Laboratory of Animal Disease and Human Health of Sichuan Province, College of Veterinary Medicine, Sichuan Agricultural University, Chengdu 611130, China; 4 Natural Medicine Research Center, College of Veterinary Medicine, Sichuan Agricultural University, Chengdu 611130, China; 5 Animal Nutrition Institute, Sichuan Agricultural University, Chengdu 611130, China

**Keywords:** Liver cancer, Polyphyllin II, Proliferation, Migration and invasion, Cofilin, AKT/NF-κB, MMP2/MMP9

## Abstract

The morbidity and mortality of primary liver cancer is one of the highest amongst all cancers. Deficiency of effective treatment and characteristics of cancer metastasis are believed to be responsible for this situation, thus a great demand is required for new agent development. Polyphyllin II (PP2), an important steroidal saponin extracted from Rhizoma Paris, has emerged as a potential anti-cancer agent, but the effects of PP2 in liver cancers and its underlying mechanisms remain unexplored. In our study, we found that PP2 could remarkably suppress the proliferation of two liver cancer cell lines, HepG2 and BEL7402, resulting in significant cell death. Besides, low doses of PP2 have displayed properties that inhibit cellular motility and invasion of liver cancer cells. In addition, we have found that PP2-mediated cofilin activity suppression was implicated in the inhibition of liver cancer cell motility. Decreased expression of two major hydrolytic enzymes (MMP2/MMP9), through the AKT/NF-κB signaling pathway may also be also responsible for this process. Rescue experiments done with either non-phosphorylatable mutant cofilin-1 (S3A) transfection or an activator of the AKT pathway significantly reversed the inhibition effects of PP2 on liver cancer cells. Taken together, we report a potential agent for liver cancer treatment and reveal its underlying mechanisms.

## Abstract (precis)

**Summary:** An important steroidal saponin extracted from Rhizoma Paris, Polyphyllin II suppressed cell viability and induced cell apoptosis in liver cancer cells. Low doses of Polyphyllin II also inhibited liver cancer cell migration and invasion.

## INTRODUCTION

Primary liver cancer is one of the most common cancers and the second leading cause of cancer-related death worldwide (Ferlay et al., 2015). Although the combined mortality rate for all cancers has been declining since 1990, the age-adjusted death rates for liver cancers increased 43% and 40% for males and females, respectively, from 2000 to 2016 in the US (Siegel et al., 2014). Chronic liver disease and cirrhosis resulting from hepatitis virus infection and excessive alcohol intake are believed to be the global leading risk factors of liver cancers (Balogh et al., 2016). Despite the development of surgical and non-surgical approaches continuously improving the overall survival rate, orthotopic liver transplantation (OLT) remains the best curative option for patients with liver cancers (Darvesh et al., 2012). However, the scarcity of donor organs precludes this therapy for most patients (Mancuso, 2013). Limited usefulness of chemotherapy or radiotherapy has been demonstrated, because they could be only applicable to patients with localized liver cancers (Darvesh et al., 2012). In addition to these problems, the metastasis of tumors has also brought great difficulty to its treatment (Christofori, 2006). Metastasis contains a multistep process wherein a primary tumor migrates from its initial site to secondary tissues/organs (Weiss, 2000), and cell migration and invasion are the key steps for cancer metastasis (Steeg, 2006). Thus, mounting efforts are needed to explore and develop more effective agents for the treatment of liver cancers, as well as for the prevention of its migration and invasion.

Recently, the extraction of anti-cancer ingredients from traditional herbs has attracted more and more interests. As a class of saponins isolated from the rhizome of a traditional Chinese herb, *Paris polyphylla* var. *yunnanensis* (so-called Rhizoma Paridis) (Liu et al., 2012), polyphyllins, have been reported to function as agents with hemostatic, analgesic, bacteriostasis, inflammatory regulation, immune modulation and especially anti-cancer properties (Shah et al., 2012; Liu et al., 2012; Zhu and Zhang, 2017; Wang et al., 2011). Lots of polyphyllins with different molecular weights have been characterized before, including polyphyllin I (PPI), polyphyllin II (PP2), polyphyllin C (PPC), polyphyllin D (PPD), polyphyllin VI (PP6) and polyphyllin VII (PP7). Li et al. have reported the inhibition of human lung cancer cells by polyphyllin I, while Shi and his colleagues found the same suppression of PPI in hepatocellular carcinoma cells (Li et al., 2016; Shi et al., 2015). PPD has been demonstrated to be effective for the inhibition of breast cancer cell proliferation both *in vitro* and *in vivo* (Lee et al., 2005), and the anti-cancer properties of PP7 were found in liver, lung, breast and colorectal cancer cells (Zhang et al., 2016a; Lin et al., 2015; He et al., 2016; Fan et al., 2015). These studies display good potential and broad application prospects for polyphyllins in anti-tumor research. According to previous studies, activated cellular apoptosis or arrested cell cycles resulting from reactive oxygen species (ROS) overproduction or autophagy were suggested to be the mechanisms underlying the anti-tumor properties of polyphyllins (Lin et al., 2015).

Polyphyllin II (PP2), also known as diosgenin-3-O-α-L-rhamnose-(1→4)–[α-L-rhamnose-(1→2)]-β-D-glucoside, is an important steroidal saponin component from Rhizoma Paridis. The anti-proliferation properties of PP2 were reported in lung cancer cells, colorectal cancer cells and ovarian cancer cells (Xiao et al., 2012; Zhang et al., 2016b; Chen et al., 2019). However, the anti-cancer activity of PP2 and its underlying mechanism against liver cancers are still unexplored and not well defined. Thus, here we aim to study the sensitivity of liver cancer cells to PP2 *in vitro*, then we will evaluate whether PP2 inhibits cell migration and invasion of liver cancer cells and further reveal the molecular mechanisms behind these processes.

## RESULTS

### PP2 suppressed the cell viability and induced cell apoptosis of HepG2 and BEL7402 cells

**Fig. 1.**
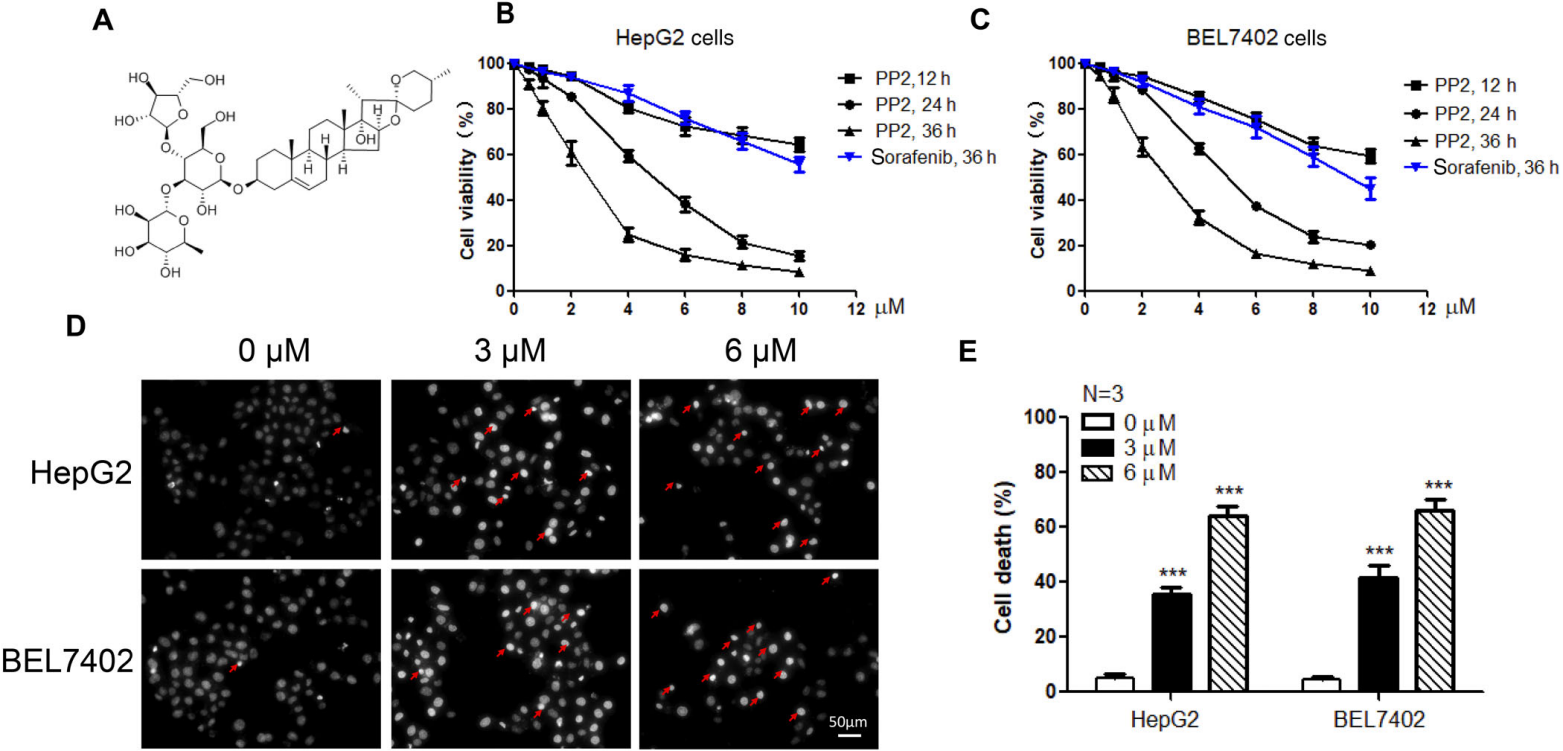
**PP2 suppressed the cell viability and induced cell apoptosis of HepG2 and BEL7402 cells.** (A) The structure of Polyphyllin II (PP2). (B,C) Quantifications show the cell viability of HepG2 (B) and BEL7402 (C) cells treated with different concentrations of PP2 for 12 h, 24 h and 36 h. Sorafenib was used as a positive control. (D) Representative images that show the nuclear morphology of HepG2 and BEL7402 cells under PP2 treatment for 24 h labeled with Hoechst 33342. Red arrows show the apoptosis cells. Scale bar: 50μm. (F) Quantification shows that PP2 induces cell death of HepG2 and BEL7402 cells according to Hoechst 33342 staining. ***P<0.001.

To evaluate the anti-proliferation property of PP2, two liver cancer cell lines (HepG2 and BEL7402) were exposed to PP2. CCK-8 assays were performed after PP2 treatment under different concentrations and times, and the results showed a dose- and time-dependent effect of PP2 on inhibitory of liver cancer cell proliferation. We found that the cell viability of both HepG2 and BEL7402 cells was suppressed by PP2 over time, and 24 h treatment was more representative of the dose dependency of PP2 (Fig. 1B,C). Sorafenib was chosen as a positive control, which is a multi-kinase inhibitor that targets c-Raf, B-Raf and VEGF receptor, and approved as systemic treatment for advanced hepatocellular carcinoma (Wei et al., 2015; Coriat et al., 2012). The 50% inhibitory concentration (IC50) values of 24 h PP2 treatment on HepG2 and BEL7402 cells were 4.8351±0.7295 μM and 4.4765±0.1611 μM, respectively. In order to figure out the reason for PP2-inhibited cellular viability, cell death was analyzed by Hoechst 33342 staining in these two cell lines after PP2 treatment (Fig. 1D). Nuclear morphology was quantified and significantly increased cell death, as quantified by the amount of nuclear pyknosis present in HepG2 and BEL7402 cells treated with 3 μM and 6 μM PP2 for 24 h (Fig. 1E). These data indicate that PP2 could induce cell death to suppress the cell viability of liver cancer cells.

### PP2 inhibited cellular motility and invasion of HepG2 and BEL7402 cells

**Fig. 2.**
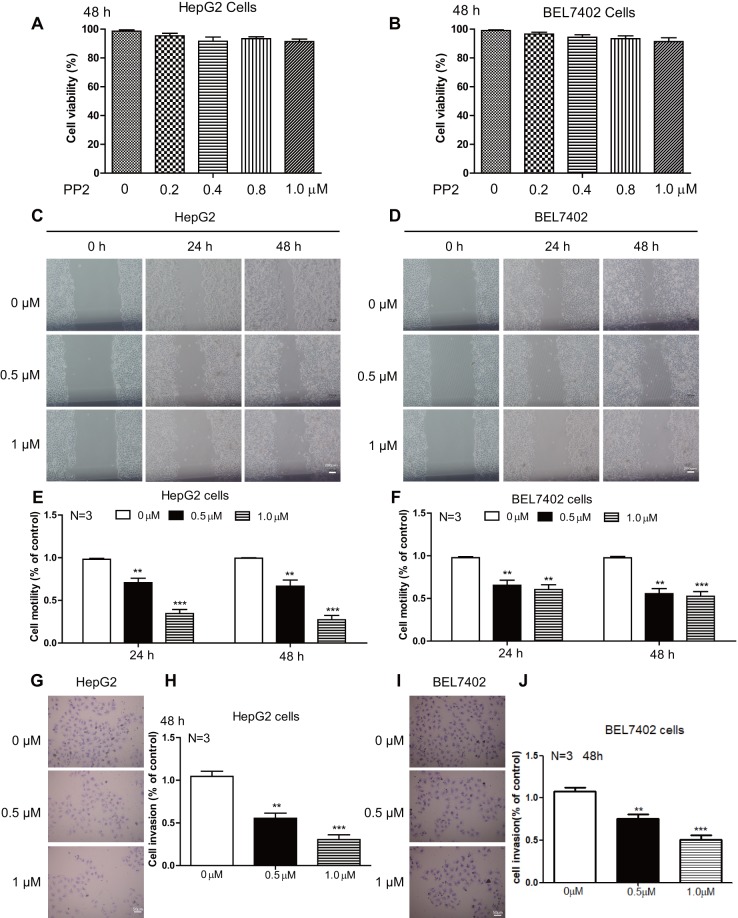
**PP2 inhibited cellular motility and invasion of HepG2 and BEL7402 cells.** (A,B) Quantifications show the cell viability of HepG2 (A) and BEL7402 (B) cells treated with low doses of PP2. (C–F) Wound healing assay (C,D) and quantifications (E,F) show decreased cellular motility of HepG2 cells and BEL7402 cells treated with 0, 0.5 and 1.0 μM PP2 for 24 and 48 h. (G,I) Cell invasion was analyzed with a Matrigel-coated Boyden chamber. Representative photomicrographs of the membrane-associated cells were assayed by 0.1% Cresyl Violet staining. (H,J) Cell invasion ability was quantitated. **P<0.01, ***P<0.001. Scale bars: 200 μm (C,D); 50 μm (G,I).

Cellular motility is important for cancer cells metastasis, as increased expressions of genes involved in cellular motility were reported to be associated with malignant tumors (Wang et al., 2005). To study the effect of PP2 on liver cancer cell motility, scratch-wound healing assays were performed with low doses of PP2 to avoid the interference of apoptosis on the results. In our work, low doses of PP2 displayed very limited cytotoxicity to HepG2 and BEL7402 cells, as shown by CCK-8 assay (Fig. 2A,B). However, 0.5 μM and 1 μM PP2 markedly suppressed the closure of the wound field gap when examined at 24 h and 48 h. Far fewer cells were observed in the wounded area in PP2-treated groups (Fig. 2C,D), and quantitative data showed significant suppression of cellular motility after PP2 treatment (Fig. 2E,F). Similarly, invasion of cancer cells is a prerequisite for tumor metastasis, so we have further performed the *in vitro* cell invasion assays to address this issue. As shown in Fig. 2G and I, 0.5 μM and 1 μM PP2 strongly inhibited cell invasion in HepG2 and BEL7402 cells, and the quantitative data indicated that the invasive abilities of HepG2 and BEL7402 cells were significantly reduced by PP2 treatment (Fig. 2H,J). These results show remarkable suppression by PP2 in migration and invasion of liver cancer cells.

### Suppressed cofilin activity was implicated in PP2-inhibited cellular motility of HepG2 and BEL7402 cells

**Fig. 3.**
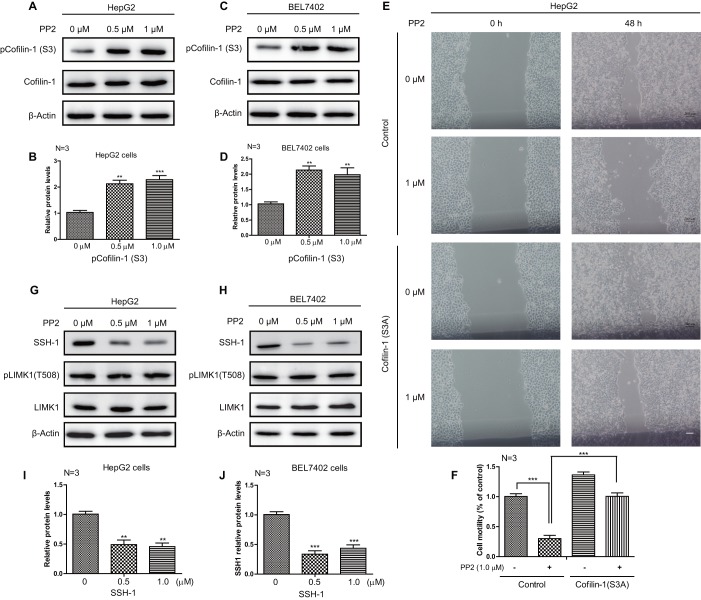
**Suppressed cofilin activity was implicated in PP2-inhibited cellular motility of HepG2 and BEL7402 cells.** (A–D) Western blots and quantifications show increased phosphorylation of cofilin-1 in the cells treated with 0, 0.5 and 1.0 μM PP2 for 24 h. (E,F) Wound healing assay and quantifications show stably expressed mutant cofilin-1 (S3A) improves cellular motility of HepG2 cells under the treatment of PP2 (1 μM). (G–J) Western blots and quantifications show decreased protein levels of SSH-1 in the cells treated with 0, 0.5 and 1.0 μM PP2 for 24 h. **P<0.01, ***P<0.001. Scale bar: 200 μm.

The dynamics of actin filament is a critical determinant of cancer cell motility, as actin polymerization can provide the forces necessary for driving the formation and extension of membrane protrusions (Wang et al., 2007). Cofilin is a ubiquitous protein, and cofilin-1 is the most abundant isoform that is able to sever actin filaments to regulate actin dynamics during cell migration (Yamaguchi and Condeelis, 2007). The activity of cofilin is highly dependent on its phosphorylation status on serine 3, which is regulated by complex processes. Generally, LIM kinase 1 (LIMK1) and its related kinases phosphorylate cofilin to suppress its actin-binding activity, while slingshot (SSH) and some other phosphatases dephosphorylate cofilin to activate its actin-binding activity (Bravo-Cordero et al., 2013). To study whether there was any correlation between PP2-suppressed cellular motility and cofilin activity, further experiments were performed. We found that PP2 could significantly increase the phosphorylation levels of cofilin-1 both in HepG2 and BEL7402 cells, indicating that cofilin activity inhibition maybe responsible for suppressed cellular motility induced by PP2 treatment (Fig. 3A–D). This hypothesis was proven by a rescue experiment that showed increased cellular motility in non-phosphorylated mutant cofilin-1 (S3A) stably expressed HepG2 cells, under the treatment of PP2 (Fig. 3E,F). The PP2-inhibited cofilin activity may result from suppressed cofilin dephosphorylation, as decreased SSH-1 protein levels were detected in PP2-treated HepG2 and BEL7402 cells (Fig. 3G–J). Meanwhile, no changes in LIMK1 were present in PP2-treated cells, indicating that PP2 did not affect the phosphorylation of cofilin (Fig. 3G–J). These data demonstrate that suppressed cofilin activity is implicated in PP2-inhibited cellular motility of HepG2 and BEL7402 cells.

### PP2 inhibited the expressions of MMP2/MMP9 and the activity of NF-κB in HepG2 and BEL7402 cells

**Fig. 4.**
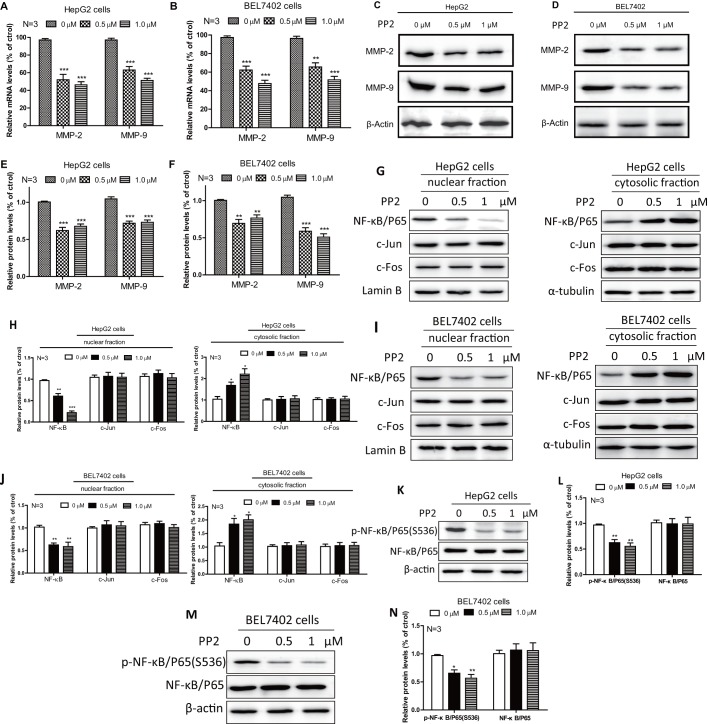
**PP2 inhibited the expression of MMP2/MMP9 and the activity of NF-κB in HepG2 and BEL7402 cells.** (A,B) The mRNA levels of MMP2/MMP9 were determined with real-time quantitative RT-PCR after the cells were incubated with 0, 0.5 and 1.0 μM PP2 for 24 h. (C–F) Western blots and quantifications show decreased protein levels of MMP2/MMP9 in the cells treated with 0, 0.5 and 1.0 μM PP2 for 24 h. (G–J) Western blots and quantifications show reduced NF-κB protein levels in the nuclear fractions and increased NF-κB protein levels in the cytosolic fractions after treating with 0, 0.5 and 1.0 μM PP2 for 24 h. (K–N) Western blots and quantifications show decreased phosphorylation of NF-κB in the cells treated with 0, 0.5 and 1.0 μM PP2 for 24 h. *P<0.05, **P<0.01, ***P<0.001.

Degradation and removal of structures in the extracellular matrix (ECM) is an essential step for cancer cells to migrate through a dense barrier of ECM (Shapiro, 1998). Matrix metalloproteinases (MMPs) are major hydrolytic enzymes degrading almost all ECM components and the basement membrane (Sounni and Noel, 2005), and previous studies have suggested that MMP2/MMP9 were closely associated with the invasion and metastasis of liver cancers (Chen et al., 2013). Thus, we have further evaluated whether inhibited MMP2/MMP9 was responsible for PP2-suppressed invasion. We found that significantly downregulated mRNA expressions of MMP2/MMP9 were present under 0.5 μM and 1 μM PP2 treatments, both in HepG2 and BEL7402 cells (Fig. 4A,B). The protein levels of MMP2/MMP9 were then analyzed by western blots (Fig. 4C,D), showing the same dose-dependent suppressed expressions as mRNA (Fig. 4E,F). Multiple transcriptional factors have been reported to be involved in the regulation of MMP2/MMP9 expression, such as c-Jun, c-Fos and NF-κB (Liu and Chang, 2010; Chen et al., 2015). Therefore, we sought to clarify whether PP2-suppressed MMP2/MMP9 resulted from abnormal expression of these factors. HepG2 and BEL7402 cells were exposed to 0.5 μM and 1 μM PP2 for 24 h, then nuclear proteins and cytosolic proteins were extracted. As shown in Fig. 3G–J, we detected remarkably reduced protein levels of NF-κB in nuclear fraction and increased protein levels of NF-κB in cytosolic fraction from both these two cell lines treated with PP2. However, no significant changes in protein levels of c-Jun or c-Fos were observed after PP2 treatment (Fig. 4G–J). In order to further show this, phosphorylation of NF-κB was quantified in whole-cell lysis by western blotting, indicating that PP2 markedly inhibited the phosphorylation of NF-κB in HepG2 and BEL7402 cells, and the protein levels of total NF-κB were not significantly changed (Fig. 4K–N). These data demonstrate that PP2-inhibited NF-κB activity may be responsible for the suppression of MMP2/MMP9 expression, which is involved in PP2-inhibited liver cancer cells metastasis.

### AKT signaling was implicated in the PP2-suppressed NF-κB/MMPs pathway

**Fig. 5.**
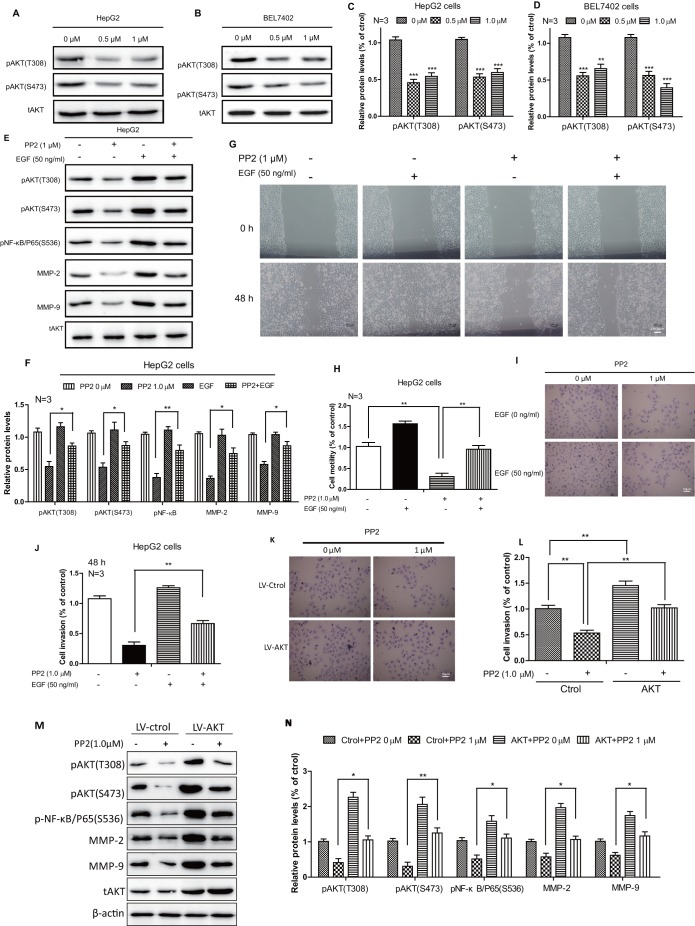
**AKT signaling was implicated in the PP2-suppressed NF-κB/MMPs pathway.** (A–D) Western blots and quantifications show reduced pAKT protein levels after treating with 0, 0.5 and 1.0 μM PP2 for 24 h in HepG2 and BEL7402 cells. (E,F) Western blots and quantification show decreased phosphorylation levels of AKT and NF-κB, as well as the expressions of MMP2/MMP9, after PP2 treatment could be rescued by growth factors. (G,H) Wound-healing assay and quantification show cellular motility after PP2 treatment could be rescued by growth factors for 48 h. (I,J) *In vitro* invasion assays and quantification show invasive ability after PP2 treatment could be rescued by growth factors for 48 h. (K,L) *In vitro* invasion assays and quantification show invasive ability after PP2 treatment for 48 h could be rescued by activated AKT. (M,N) Western blots and quantification show decreased phosphorylation levels of AKT and NF-κB, as well as the expressions of MMP2/MMP9, after PP2 treatment could be rescued by activated AKT.*P<0.05, **P<0.01, ***P<0.001. Scale bars: 200 μm (G); 50 μm (I,K).

AKT signaling was believed to be a central player in tumorigenesis, in migration as well as invasion (Kim et al., 2001). Previous studies have reported that AKT can phosphorylate IkappaB, which in turn degrades it through the ubiquitination pathway. This further leads to the nuclear translocation and activation of NF-κB (Ozes et al., 1999). Given that PP2 inhibited the activity of NF-κB, we evaluated the effects of PP2 on AKT activity. We found that 0.5 μM and 1 μM PP2 significantly suppressed the phosphorylation of AKT in HepG2 and BEL7402 cells (Fig. 5A–D). In addition, rescue studies were performed in PP2-treated HepG2 cells with epidermal growth factor (EGF), which specifically activates AKT signaling. Increased protein levels of phosphorylation AKT, phosphorylation NF-κB, as well as MMP2/MMP9 were detected in the PP2 and EGF combined group compared with PP2-treated group (Fig. 5E,F). Moreover, we further observed increased cellular motility (Fig. 5G,H) and invasive ability (Fig. 5I,J) through scratch-wound healing and transwell invasion assays in the EGF-rescued group compared with the PP2-treated group. Furthermore, the HepG2 stable cell line that expressed activated AKT (a double mutation S473D and T308D) (Nguyen and Mitchell, 2013) was generated by lentiviral particles. AKT signaling was activated in AKT stable HepG2 cells. We further performed *in vitro* cell invasion assays to discuss the relationship between PP2 and AKT-mediated liver cancer cell invasion. As shown in Fig. 5K and L, we observed increased cell invasive ability in the AKT group compared with the control group after treatment with PP2. Increased protein levels of phosphorylation AKT, phosphorylation NF-κB, as well as MMP2/MMP9 were detected in AKT stable HepG2 cells compared with control HepG2 cells after treatment with PP2 (Fig. 5M,N). These data suggest a direct relationship between PP2 and AKT/NF-κB-mediated liver cancer cell migration and invasion.

## DISCUSSION

Rhizoma Paridis is a traditional Chinese herb that is widely used for antifebrile, alexipharmic, detumescent, demulcent, hemostatic and hepatopathy treatments (Wu et al., 2004). Modern pharmacology studies have further expanded its application in cancer treatments, and steroidal saponins were reported to be its main anti-cancer components (Sun et al., 2007). Lots of steroidal saponins have been isolated and characterized by previous studies, and preliminary studies of anti-proliferation effects on liver cancer of these saponins were present. Liu et al. found that total extraction of Rhizoma Paridis saponins displayed remarkable inhibition of diethylnitrosamine-induced liver cancer in rats (Liu et al., 2016), and Man et al. demonstrated similar suppression of liver cancer of this total extraction in H22 hepatocarcinoma mice (Man et al., 2014). Besides, *in vitro* experiments with HepG2 cells have also shown this inhibition effect of Rhizoma Paridis total saponins (Cheng et al., 2008). For the individual monomeric saponin from Rhizoma Paridis, PPI and PP7 were reported to inhibit the growth of hepatoma xenografts in nude mice (Chen et al., 2014) and the proliferation of HepG2 cells (Long et al., 2015). However, the effect of PP2 on liver cancers is still unexplored and not well defined. In our study, we have for the first time demonstrated that high doses of PP2 could significantly induce cell death in HepG2 and BEL7402 cells and suppress their proliferation. Meanwhile, suppressed migration and invasion were observed in HepG2 and BEL7402 cells when they were exposed to low doses of PP2. These results suggest a broad prospect for PP2 in liver cancer treatment.

Cofilin is widely reported to be critical for cell motility by regulating actin dynamics at lamellopodia that are involved in cell invasion (Charras and Paluch, 2008). However, controversial studies have been published about whether cofilin increased or suppressed cancer cell invasion. On the one hand, the expression of cofilin is upregulated in some highly invasive cancer cells, such as C6 rat glioblastoma cells, A549 human lung cancer cells and human pancreatic cancer cells, but downregulation is also observed in other cancer cells with high metastatic potential, such as hepatocellular carcinoma (HCC) cells and ovarian surface epithelium (OSE) cells (Kanellos and Frame, 2016; Wang et al., 2007). On the other hand, overexpression of the wild-type or non-phosphorylatable cofilin mutant S3A increases cell invasion of melanoma cells and human glioblastoma cells, but inhibits the invasiveness of human lung cancer (H1299) cells by disrupting the actin cytoskeleton at the leading edge of the cell (Jang et al., 2012; Wang et al., 2007; Yamaguchi and Condeelis, 2007). These results suggest that a balanced contribution of cofilin expression and activity is required for cancer cell motility. In our study, we found that PP2 could suppress the activity of cofilin by enhancing its phosphorylation, which resulted from decreased SSH-mediated dephosphorylation but not increased LIMK1-mediated phosphorylation of cofilin. This displays the function of PP2 in regulating the activity balance of cofilin to suppress the cellular motility of liver cancer cells.

Abnormal expressions of MMP2/MMP9 were often observed in tumor tissues or cancer cell lines and were associated with tumor metastasis in many cancers including liver cancer (Roomi et al., 2009). In our study, we found that PP2 treatment in liver cancer cells suppressed cell migration and invasion, accompanied by downregulated expression of MMP2/MMP9, suggesting they were responsible for the suppression effect of PP2. Furthermore, by using rescue experiments, we demonstrated that PP2-inhibited MMP2/MMP9 was dependent on the PI3K/AKT/NF-κB pathway. This results are consistent with the work done by Chen et al. (2019). They found that PP2 suppresses the activity of the NF-κB signaling pathway and inhibits colorectal carcinogenesis. Another work from Zhang, et al. has reported that inhibited phosphorylation of AKT was induced by PP2 in lung cancer cells, which was also present in PP2-treated liver cancer cells from our study (Zhang et al., 2016b). PP2 has been demonstrated to modify ERK1/2 signals in ovarian cancer cells, but we have not detected any effects of PP2 in this regard (data not shown) (Xiao et al., 2012). These results suggest different underlying mechanisms of PP2 on different cancer treatments.

In summary, we have provided evidence that PP2 capably suppresses the proliferation of liver cancer cells induced by cell death. We further revealed inhibited liver cancer cell migration and invasion through suppressed cofilin activity, as well as downregulation of the AKT/NF-κB signaling pathway resulted in a decrease MMP-2/MMP-9 expression by PP2 treatment. These findings suggest that PP2 is a potential anti-invasion and anti-metastasis agent for the treatment of liver cancers.

## MATERIALS AND METHODS

### Reagents

PP2 was purchased from Chengdu Must Bio-Technology Co., Ltd. (Chengdu, China) and the purity of PP2 was ≥98%. Sorafenib was purchased from Sigma-Aldrich. The Cell Counting Kit-8 (CCK-8) was purchased from Dojindo Molecular Technologie (Kumamoto, Japan). Hoechst Staining Kit and Crystal Violet Staining Solution were purchased from Beyotime Institute of Biotechnology (Haimen, China). Epidermal growth factor (EGF) was purchased from PeproTech (Rocky Hill, USA). The lentiviral particles {control lentiviral particles, lentiviral particles expressed active cofilin1 protein [cofilin (S3A)] and lentiviral particles expressed activated AKT1 protein [a double mutation S473D and T308D]} were obtained from Genechem Group (Shanghai, China).

### Cell culture

The liver cancer cell lines HepG2 and BEL7402 were obtained from the Shanghai Institutes of Biological Sciences, Chinese Academy of Sciences (Shanghai, China). HepG2 and BEL7402 cells were cultured in DMEM with 10% fetal bovine serum (Gibco; Thermo Fisher Scientific) and 1% penicillin–streptomycin (Invitrogen; Thermo Fisher Scientific). HepG2 stable cell line that expressed active cofilin1 protein [cofilin (S3A), a point mutation targeting the Ser 3 phosphorylation] and HepG2 stable cell line that expressed activated AKT1 (a double mutation S473D and T308D) were generated by lentiviral particles. Briefly, HepG2 cells were seeded into 12-well plates with a density of 5×10^4^. After incubation overnight, the control lentiviral particles and cofilin-1 (S3A) lentiviral particles were added into corresponding wells. Seventy-two hours after transduction, cells were cultured in the presence of puromycin to select cells with successful transduction. We successfully generated control HepG2 stable cell line and cofilin-1 (S3A) HepG2 stable cell line. AKT stable HepG2 cell line was successfully generated as the cofilin-1 (S3A) HepG2 stable cell line. All cells were maintained at 37°C with 5% CO_2_.

### Cell viability assay

Cell viability was determined by CCK-8 assay. The cells were seeded with a density of 5×10^3^ cells/well in 96-well plates and incubated overnight in DMEM with 10% FBS at 37°C. Then, cells were treated with different concentrations of PP2 for 12 h, 24 h and 36 h. CCK-8 was added to each well of the 96-well plate (CCK-8, 10 μl/well), followed by further incubation for 1 h. The absorbance was measured at 450 nm using a microplate reader (Thermo Fisher Scientific). The cell viability rate was calculated according to the following formula:
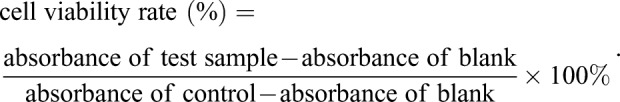


### Hoechst staining

Briefly, HepG2 and BEL7402 cells were plated in 12-well plates with a density of 5×10^4^ cells/well and incubated overnight in DMEM with 10% FBS at 37°C. Then cells were treated with different concentrations of PP2 for 24 h. Next, the cells were washed in PBS three times and incubated in Hoechst 33342 staining solution (10 μg/ml) for 30 min at 4°C. Finally, fluorescence microscopy was preformed to observe the nuclear changes of HepG2 and BEL7402 cells. For each treatment group ≥1000 cells were analyzed in triplicate.

### Wound-healing assay

Briefly, HepG2 and BEL7402 cells were plated in six-well plates. When HepG2 and BEL7402 cells were grown to a 90% confluent monolayer, scratched with a P-200 pipette tip to create wounds and washed three times to remove shed cells. Then the cells were incubated in DMEM (no serum) with 0, 0.5 and 1.0 μM of PP2 for 24 h and 48 h. Phase contrast images were taken by a microscopy system. The width of the scratches were measured by using ImageJ software, cell motility was calculated according to the following formula:
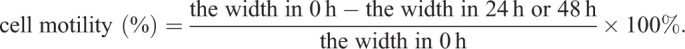


### *In vitro* cell invasion assay

Cell invasion was performed by transwell plate method. Briefly, HepG2 and BEL7402 cells were resuspended in serum-free DMEM, and 500 μl of cell suspension (5×10^5^ cells/ml) were seeded into 12-well plate transwell inserts (8-μm pore size polycarbonate membrane) that were pre-coated with Matrigel. Lower chambers were filled with 1 ml DMEM (10% FBS) and different concentrations of PP2 were added into the top chamber. After 48 h incubation, the cells on the upper surface of the membrane were removed with cotton swabs, and the cells that invaded the lower surface of the membrane were fixed with 4% paraformaldehyde and stained with 0.1% Cresyl Violet solution. Phase contrast images were taken by a microscopy system. The numbers of cells that invaded the lower surface of the membrane were quantitated.

### RT-PCR

The total RNA of HepG2 and BEL7402 cells were extracted by using Trizol reagent (Invitrogen; Thermo Fisher Scientific), according to the manufacturer's instructions after being treated with the indicated concentrations of PP2. All RNAs were reverse-transcribed into cDNA using a PrimeScript RT Master Mix kit (Takara Biotechnology, Co., Ltd, Dalian, China). RT-PCR was performed using the SYBR Premix Ex Taq. II kit (Takara Biotechnology, Co., Ltd, Dalian, China). Relative fold-changes were determined using GAPDH genes as normalizer control. Primer sequences used were as follows: MMP-2 mRNA forward, 5′-TGA GCT CCC GGA AAA GAT TG-3′ and reverse, 5′-TCA GCA GCC TAG CCA GTCG-3′; MMP-9 mRNA forward, 5′-TCC CTG GAG ACC TGA GAA CC-3′ and reverse, 5′-CGG CAA GTC TTC CGA GTA GTT-3′; GAPDH mRNA forward, 5′-GGA GCG AGA TCC CTC CAA AAT-3′ and reverse, 5′-GGC TGT TGT CAT ACT TCT CAT GG-3′.

### Western blotting analysis

Western blotting was performed according to standard procedures. Briefly, to extract protein from HepG2 and BEL7402 cells, which were sonicated in lysis buffer (2% SDS with proteinase inhibitors and phosphatase inhibitor). The protein concentration of each sample was measured using the BCA Protein Assay kit (Thermo Fisher Scientific Pierce). Equal amounts of protein were loaded into each lane of a gel and separated by SDS-PAGE. The proteins were transferred onto PVDF membranes using standard procedures. The membranes were then blocked with 5% non-fat dry milk in TBST (TBS with 0.1% Tween 20, pH 7.6) for 1 h at room temperature (RT), incubated with primary antibodies overnight at 4°C and incubated with appropriate secondary antibodies for 1 h at RT. Finally, proteins were detected with ECL reagent (Thermo Fisher Scientific Pierce) and the membranes were exposed to film (Kodak). The antibodies in our experiments include: AKT (Cell Signaling Technology, 9272S, 1:2000 dilution), pAKT (S473) (Cell Signaling Technology, 4060S, 1:1000 dilution), pAKT (T308) (Cell Signaling Technology, 2965S, 1:1000 dilution), MMP-2 (Cell Signaling Technology, 40994, 1:1000 dilution), MMP-9 (Cell Signaling Technology, 13677, 1:1000 dilution), NF-κB/P65 (Cell Signaling Technology, 8242, 1:1000 dilution), c-Jun (Cell Signaling Technology, 9165, 1:500 dilution), c-Fos (Cell Signaling Technology, 2250, 1:500 dilution), Lamin B1 (Cell Signaling Technology, 13435, 1:1000 dilution), p-NF-κB/P65 (S536) (Abcam, ab28856, 1:1000 dilution), Cofilin (Abcam, ab42824, 1:1000 dilution), pCofilin (Abcam, ab12866, 1:1000 dilution), LIMK1 (Abcam, ab108507, 1:1000 dilution), pLIMK1 (T508) (Abcam, ab108507, 1:1000 dilution), SSH1 (Abcam, ab107799, 1:500 dilution), β-Actin (Boster, BM0627, 1:2000 dilution), Goat anti-Rabbit IgG (H+L) secondary antibody (Pierce, 31460, 1:10,000 dilution) and Goat anti-Mouse IgG (H+L) secondary antibody (Pierce, 31430, 1:10,000 dilution).

### Statistical analysis

Statistical evaluation was conducted using SPSS 19.0 (SPSS, Inc., Chicago, IL, USA). All data are presented as the mean±s.d. Differences among multiple groups were compared by one-way analysis of variance, and differences between two groups were compared by the Student's *t*-test. *P*<0.05 was considered to indicate a statistically significant difference.
