# Correction: Polyphyllin II inhibits liver cancer cell proliferation, migration and invasion through downregulated cofilin activity and the AKT/NF-κB pathway

**DOI:** 10.1242/bio.059765

**Published:** 2023-01-12

**Authors:** Dejiang Pang, Chengcheng Yang, Chao Li, Yuanfeng Zou, Bin Feng, Lixia Li, Wentao Liu, Qihui Luo, Zhengli Chen, Chao Huang

There were errors published in *Biol. Open* (2020) **9**, bio046854 (doi:10.1242/bio.046854).

In Fig. 2, the results from the HepG2 cells (G,H) and the BEL7402 cells (I,J) were mistakenly swapped. In addition, the control panel from Fig. 2I was repeated in Fig. 5I. A replicate control panel has been substituted.

**Figure:**
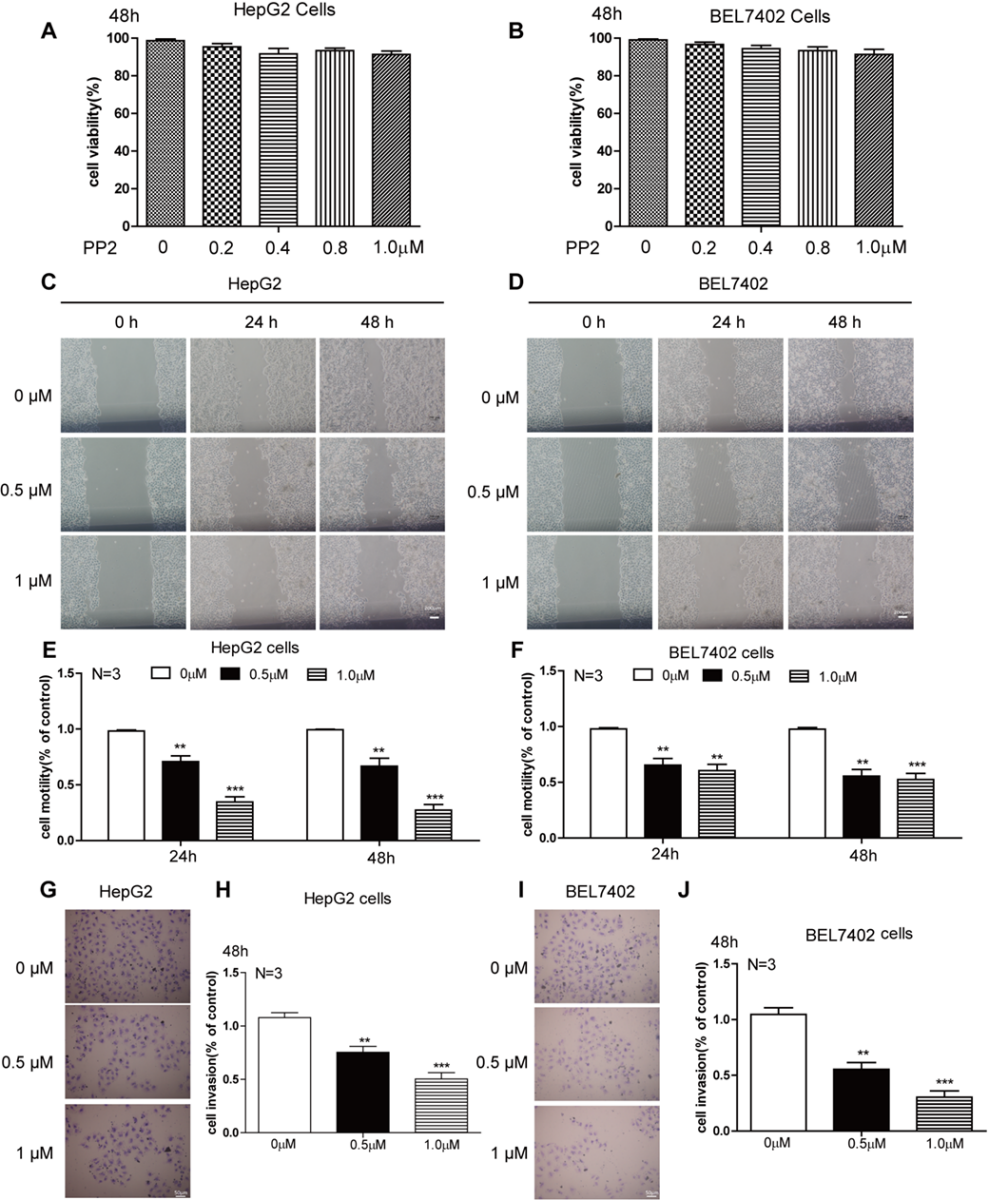
**Fig. 2 (corrected). PP2 inhibited cellular motility and invasion of HepG2 and BEL7402 cells.** (A,B) Quantifications show the cell viability of HepG2 (A) and BEL7402 (B) cells treated with low doses of PP2. (C–F) Wound healing assay (C,D) and quantifications (E,F) show decreased cellular motility of HepG2 cells and BEL7402 cells treated with 0, 0.5 and 1.0 μM PP2 for 24 and 48 h. (G,I) Cell invasion was analyzed with a Matrigel-coated Boyden chamber. Representative photomicrographs of the membrane-associated cells were assayed by 0.1% Cresyl Violet staining. (H,J) Cell invasion ability was quantitated. **P<0.01, ***P<0.001. Scale bars: 200 μm (C,D); 50 μm (G,I).

**Figure:**
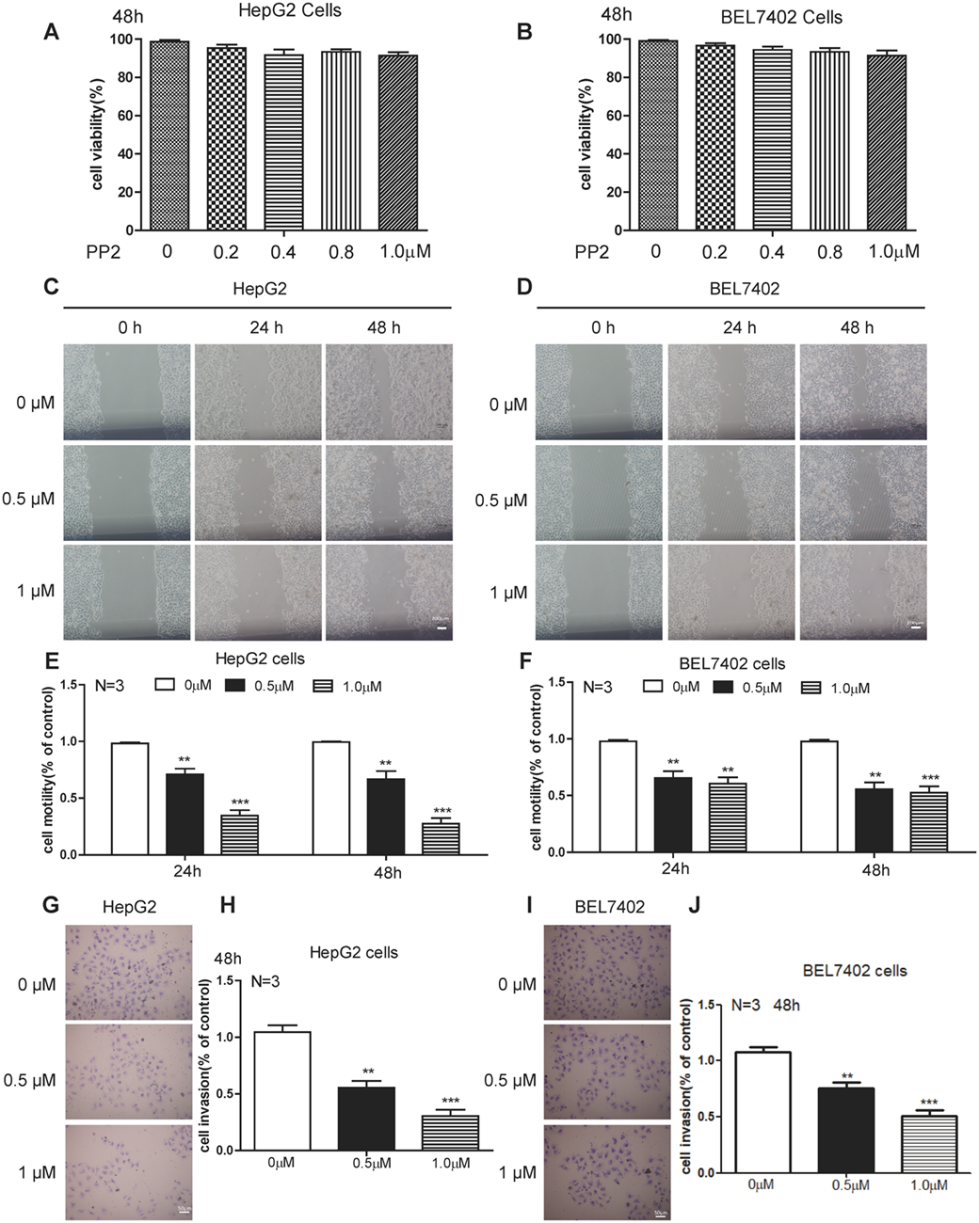
**Fig. 2 (original). PP2 inhibited cellular motility and invasion of HepG2 and BEL7402 cells.** (A,B) Quantifications show the cell viability of HepG2 (A) and BEL7402 (B) cells treated with low doses of PP2. (C–F) Wound healing assay (C,D) and quantifications (E,F) show decreased cellular motility of HepG2 cells and BEL7402 cells treated with 0, 0.5 and 1.0 μM PP2 for 24 and 48 h. (G,I) Cell invasion was analyzed with a Matrigel-coated Boyden chamber. Representative photomicrographs of the membrane-associated cells were assayed by 0.1% Cresyl Violet staining. (H,J) Cell invasion ability was quantitated. **P<0.01, ***P<0.001. Scale bars: 200 μm (C,D); 50 μm (G,I).

**Figure:**
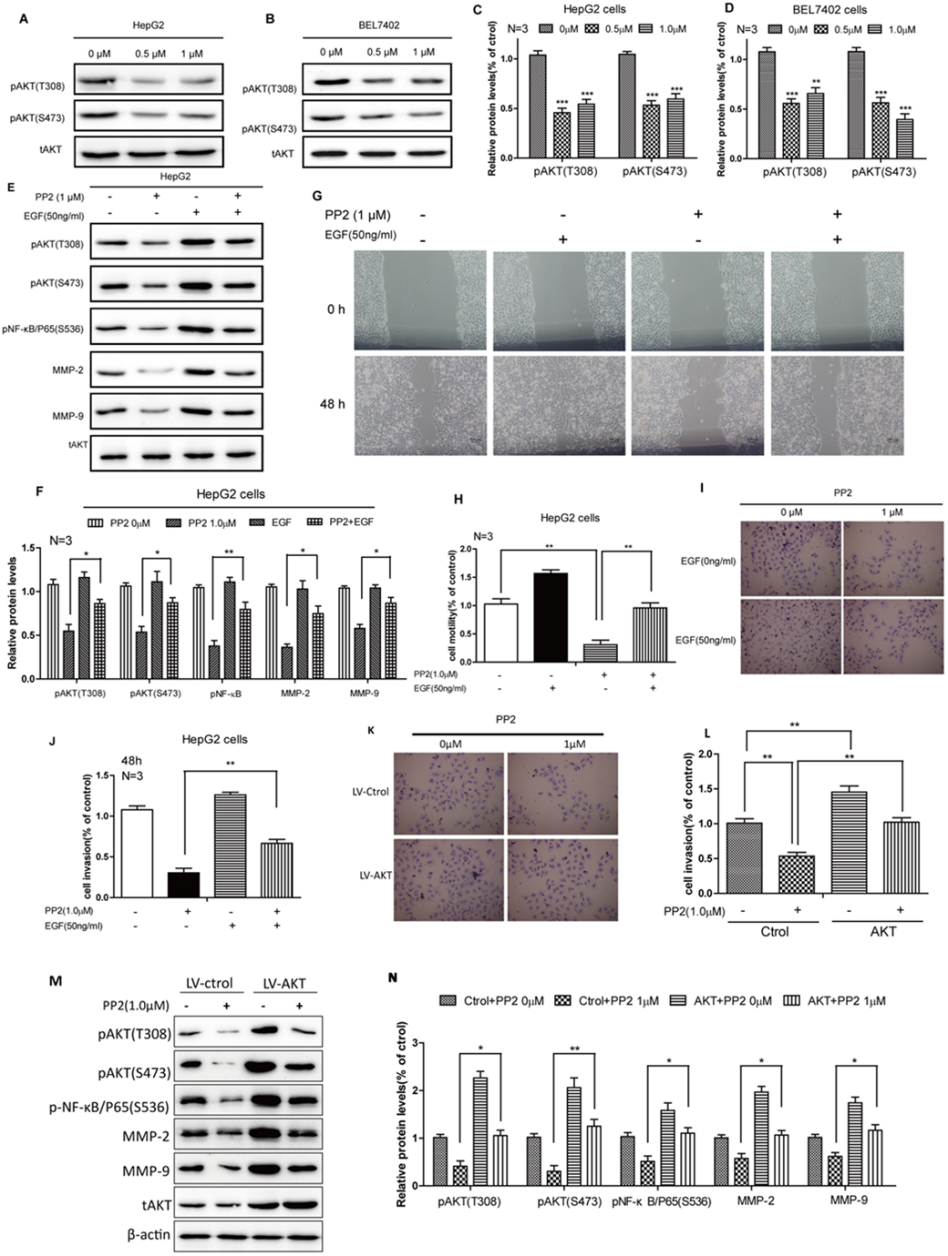
**Fig. 5 (corrected). AKT signaling was implicated in the PP2-suppressed NF-κB/MMPs pathway.** (A-D) Western blots and quantifications show reduced pAKT protein levels after treating with 0, 0.5 and 1.0 μM PP2 for 24 h in HepG2 and BEL7402 cells. (E,F) Western blots and quantification show decreased phosphorylation levels of AKT and NF-κB, as well as the expressions of MMP2/MMP9, after PP2 treatment could be rescued by growth factors. (G,H) Wound-healing assay and quantification show cellular motility after PP2 treatment could be rescued by growth factors for 48 h. (I,J) *In vitro* invasion assays and quantification show invasive ability after PP2 treatment could be rescued by growth factors for 48 h. (K,L) *In vitro* invasion assays and quantification show invasive ability after PP2 treatment for 48 h could be rescued by activated AKT. (M,N) Western blots and quantification show decreased phosphorylation levels of AKT and NF-κB, as well as the expressions of MMP2/MMP9, after PP2 treatment could be rescued by activated AKT. *P<0.05, **P<0.01, ***P<0.001. Scale bars: 200 μm (G); 50 μm (I,K).

**Figure:**
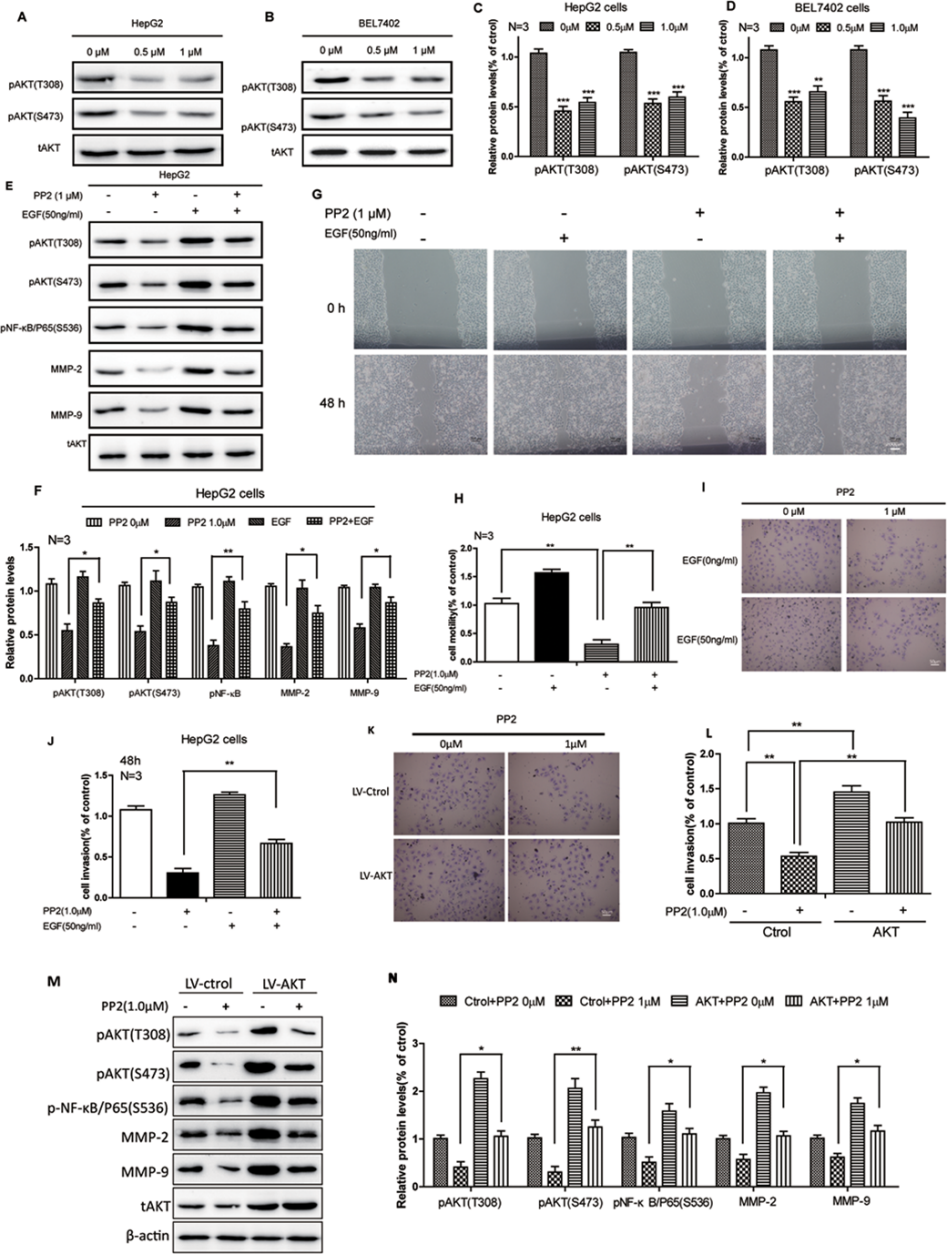
**Fig. 5 (original). AKT signaling was implicated in the PP2-suppressed NF-κB/MMPs pathway.** (A-D) Western blots and quantifications show reduced pAKT protein levels after treating with 0, 0.5 and 1.0 μM PP2 for 24 h in HepG2 and BEL7402 cells. (E,F) Western blots and quantification show decreased phosphorylation levels of AKT and NF-κB, as well as the expressions of MMP2/MMP9, after PP2 treatment could be rescued by growth factors. (G,H) Wound-healing assay and quantification show cellular motility after PP2 treatment could be rescued by growth factors for 48 h. (I,J) *In vitro* invasion assays and quantification show invasive ability after PP2 treatment could be rescued by growth factors for 48 h. (K,L) *In vitro* invasion assays and quantification show invasive ability after PP2 treatment for 48 h could be rescued by activated AKT. (M,N) Western blots and quantification show decreased phosphorylation levels of AKT and NF-κB, as well as the expressions of MMP2/MMP9, after PP2 treatment could be rescued by activated AKT. *P<0.05, **P<0.01, ***P<0.001. Scale bars: 200 μm (G); 50 μm (I,K).

The corrected and original figures are shown below. Both the online full-text and PDF versions of the article have been updated. The authors apologise to readers for this error, which does not impact the results or conclusions of this paper.

